# First Case of Erythema Nodosum Associated With Pfizer Vaccine

**DOI:** 10.7759/cureus.19529

**Published:** 2021-11-13

**Authors:** Mohammed H Aly, Abdulrahman A Alshehri, Abdelgaffar Mohammed, Abdulrahman M Almalki, Walaa A Ahmed, Alhanouf M Almuflihi, Atheer A Alwafi

**Affiliations:** 1 Department of Internal Medicine, Security Forces Hospital, Makkah, SAU; 2 Department of Rheumatology, Security Forces Hospital, Makkah, SAU; 3 Department of Medicine, College of Medicine, Umm Al-Qura University, Al-Abdia Main Campus, Makkah, SAU

**Keywords:** pfizer, vaccines, cutaneous side effects, erythema nodosum, covid-19

## Abstract

Vaccine-related erythema nodosum is uncommon, especially after the coronavirus disease 2019 (COVID-19) vaccine. This study presents the first case of the Pfizer vaccine associated with erythema nodosum. A 22-year-old healthy woman presented with a five-day history of several red painful areas with swelling in the lower extremities that started one day after receiving the first dose of Pfizer vaccine. Past medical history, laboratory investigation, and chest radiograph revealed normal results. Erythema nodosum is an immune reaction that manifests as multiple, painful nodules commonly seen on the shin that resolved spontaneously two to eight weeks after the onset. In the absence of laboratory and chest radiograph abnormalities and shortly after receiving the Pfizer vaccine, vaccine-related erythema nodosum is the only possible explanation.

## Introduction

Severe acute respiratory syndrome coronavirus 2 (SARS-CoV-2) pneumonia was first reported in 2019 then declared as a pandemic in 2020 which is also known as coronavirus disease 2019 (COVID-19) [1]. Different types of vaccines have been developed since the start of the COVID-19 as an emergency response to the pandemic which includes Pfizer COVID-19 vaccine (BNT162b2), AstraZeneca/Oxford COVID-19 vaccine also known as ChAdOx1nCoV-19 (AZD1222), Johnson & Johnson vaccine (Ad26.COV2.S), and Moderna COVID-19 vaccine (mRNA-1273) [2]. Erythema nodosum is a hypersensitivity reaction that causes inflammation of the subcutaneous tissue due to immune complex deposition. One to five per 100,000 individuals worldwide experience erythema nodosum which is commonly seen in age groups between 18-34 years with female to male predominance 3-5:1. Fifty percent of the reported cases of erythema nodosum are idiopathic. In addition, it is commonly associated with infections, inflammatory bowel disease, medications, sarcoidosis, and pregnancy. However, erythema nodosum related to the vaccine is rarely seen [3]. Herein, we present a case of a 22-year-old healthy woman who presented with erythema nodosum after receiving the Pfizer COVID-19 vaccine.

## Case presentation

**Figure 1:**
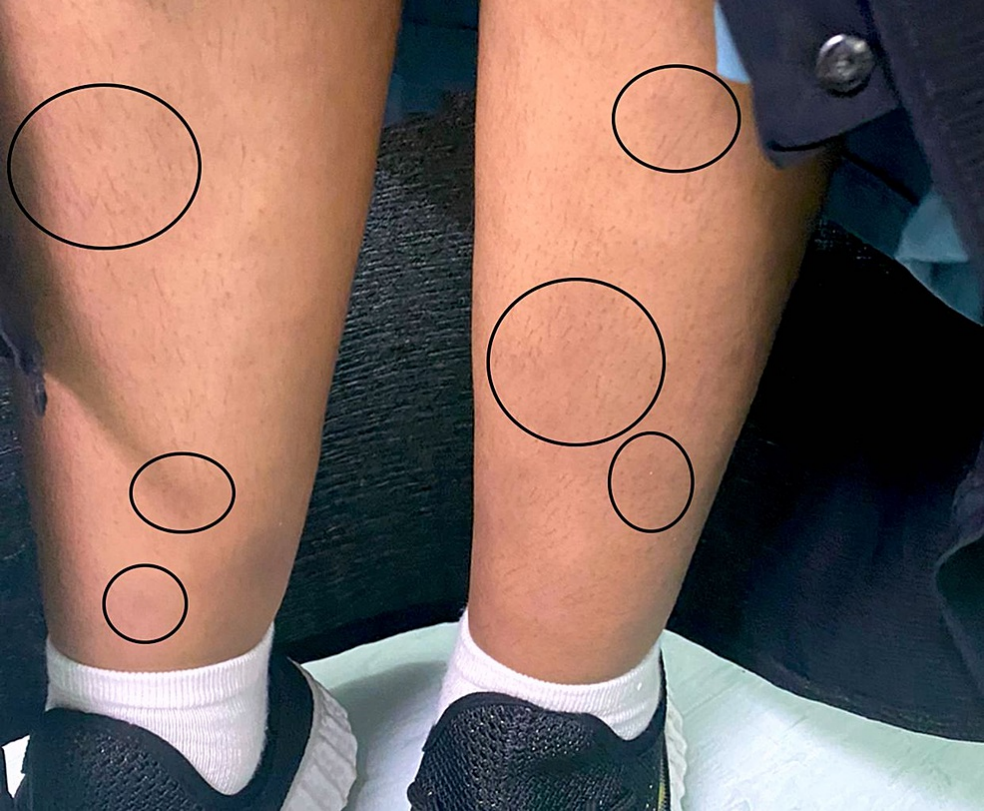
The image represents the flattened nodules over the patient’s lower extremities after receiving the treatment for three days.

A 22-year-old healthy woman presented to the emergency department complaining of a five-day history of several red and painful areas that were associated with swelling of the lower extremities. Her complaint started one day after receiving the first dose of the Pfizer vaccine. She had received all vaccinations during childhood without any complications. There is no history of preceding infections including COVID-19 infection, sore throat, or diarrhea. In addition, she never complained of prior skin lesions or systemic diseases. She did not use any topical or systemic medication. All the laboratory investigations were normal. She stated that there is no history of contact with the sick patients and no family history of tuberculosis. Skin examination revealed multiple, tender, and erythematous nodules were present on her both legs. Based on the patient history, examination, and laboratory investigation all pointed to the vaccine-related erythema nodosum; however, we considered the Arthus-like phenomenon which can mimic the erythema nodosum lesions. She received oral ibuprofen 600 mg four times daily to relieve symptoms. Three days after receiving the treatment, she improved and the nodules started to flatten (Figure 1). Two weeks later, on the follow-up examination, her symptoms had completely resolved.

## Discussion

Vaccines are meant to be protective against infectious diseases, in which it stimulates the host’s immune system through increasing the antigen-specific memory cells that eventually decreases the infection effects. However, it can induce some side effects [4]. There are four types of COVID-19 vaccines - Pfizer vaccine, AstraZeneca/Oxford vaccine, Johnson & Johnson vaccine, and Moderna [2]. Pfizer vaccine/BNT162b2 exhibits 95% efficacy in preventing the illness but showed some common systemic adverse effects that were reported in the clinical trials, such as fever, headache, fatigue, and lymphadenopathy. Other local adverse effects were reported such as pain at the site of injection, redness, and swelling [5]. Erythema nodosum is a sudden immune reaction that subsides spontaneously (two to eight weeks), manifests as multiple tender erythematous subcutaneous nodules, most commonly affect the pretibial area, 98% limited to the shins, and rarely seen on other areas. Although half of the reported cases are idiopathic, erythema nodosum is associated with other conditions such as infections, inflammatory bowel disease, medications, sarcoidosis, autoimmune diseases, paraneoplastic syndrome, pregnancy, and rarely seen post-vaccination. The diagnosis is clinical, and biopsy is not necessary. Nevertheless, a complete laboratory evaluation that includes a complete blood count (CBC) with differential erythrocyte sedimentation rate (ESR), C-reactive protein (CRP), throat swab culture and, antistreptolysin O (ASO) titers, and a chest radiograph is needed to figure out the underlying cause [3]. According to a previous study, eight vaccines were found to be related to erythema nodosum, in which they reported the following bacille-Calmette-Guerin vaccine, hepatitis B vaccine, human papillomavirus vaccine, malaria vaccine, rabies vaccine, smallpox vaccine, tetanus, diphtheria, and pertussis vaccine, and typhoid and cholera vaccine [6]. In addition, our literature review revealed three reported cases of COVID-19 vaccine-induce erythema nodosum, in which two of them were AstraZeneca/Oxford vaccines [7,8] and the other one was Medigen vaccine (MVC-COV1901) (Table 1) [9].

**Table 1:** Documented vaccines related to the erythema nodosum. COVID-19: coronavirus disease 2019

No.	Vaccine	Reference
1	Bacille-Calmette-Guerin	[10-12]
2	Hepatitis B	[13-15]
3	Human papillomavirus	[16]
4	Malaria	[17]
5	Rabies	[18,19]
6	Smallpox	[20]
7	Tetanus, diphtheria, and pertussis	[6]
8	Typhoid and cholera	[21]
9	AstraZeneca/Oxford COVID-19 vaccines	[7,8]
10	Medigen COVID-19 vaccine	[9]
11	Pfizer COVID-19 vaccine	This case

Our case is a 22-year-old healthy woman who developed erythema nodosum manifestations shortly after receiving the Pfizer vaccine with no past medical history and normal laboratory and chest radiograph which suggests that erythema nodosum was related only to the Pfizer vaccine.

## Conclusions

Vaccine-related erythema nodosum is an uncommon condition, especially with COVID-19 vaccines. The current study reported the first case of erythema nodosum related to the Pfizer vaccine. This study aimed to raise the recognition in such conditions, in the absence of laboratory and chest radiograph abnormalities and shortly after receiving the Pfizer vaccine, vaccine-related erythema nodosum is the only possible explanation. We recommend further studies with a higher level of evidence to investigate and assess the relation of this condition with the vaccine.

## References

[1] (2020). Listings of WHO’s response to COVID-19 [Internet]. https://www.who.int/news/item/29-06-2020-covidtimeline.

[2] Francis AI, Ghany S, Gilkes T, Umakanthan S (2021). Review of COVID-19 vaccine subtypes, efficacy and geographical distributions. Postgrad Med J.

[3] Leung AK, Leong KF, Lam JM (2018). Erythema nodosum. World J Pediatr.

[4] Megha KB, Nayar SA, Mohanan PV (2021). Vaccine and vaccination as a part of human life: in view of Covid-19. Biotechnol J.

[5] Polack FP, Thomas SJ, Kitchin N (2020). Safety and efficacy of the BNT162b2 mRNA Covid-19 vaccine. N Engl J Med.

[6] Cohen PR (2013). Combined reduced-antigen content tetanus, diphtheria, and acellular pertussis (tdap) vaccine-related erythema nodosum: case report and review of vaccine-associated erythema nodosum. Dermatol Ther (Heidelb).

[7] Cameli N, Silvestri M, Mariano M, Bennardo L, Nisticò SP, Cristaudo A (2021). Erythema nodosum following the first dose of ChAdOx1-S nCoV-19 vaccine. J Eur Acad Dermatol Venereol.

[8] Mehta H, Handa S, Malhotra P (2021). Erythema nodosum, zoster duplex and pityriasis rosea as possible cutaneous adverse effects of Oxford-AstraZeneca COVID-19 vaccine: report of three cases from India. J Eur Acad Dermatol Venereol.

[9] Hsu HT, Su HA, Chen YC (2021). Erythema nodosum, after Medigen vaccination against COVID-19?. J Formos Med Assoc.

[10] Franco-Paredes C, Díaz-Borjon A, Senger MA, Barragan L, Leonard M (2006). The ever-expanding association between rheumatologic diseases and tuberculosis. Am J Med.

[11] Busselo IS, Vergara EO, Pérez-Yarza EG, Palma FL, Benito AR, Andrade YA (2004). Erythema nodosum: etiological changes in the last two decades. [Article in Spanish]. An Pediatr (Barc).

[12] Galzerano G, Sorrentini R (1958). Case of erythema nodosum appearing after BCG vaccination. [Article in Italian]. Arch Tisiol Mal Appar Respir.

[13] Rogerson SJ, Nye FJ (1990). Hepatitis B vaccine associated with erythema nodosum and polyarthritis. BMJ.

[14] Goolsby PL (1989). Erythema nodosum after Recombivax HB hepatitis B vaccine. N Engl J Med.

[15] Di Giusto CA, Bernhard JD (1986). Erythema nodosum provoked by hepatitis B vaccine. Lancet.

[16] Longueville C, Doffoel-Hantz V, Hantz S, Souyri N, Nouaille Y, Bédane C, Sparsa A (2012). Gardasil®-induced erythema nodosum. [Article in French]. Rev Med Interne.

[17] Wu Y, Ellis RD, Shaffer D (2008). Phase 1 trial of malaria transmission blocking vaccine candidates Pfs25 and Pvs25 formulated with montanide ISA 51. PLoS One.

[18] Kaliyadan F, Dharmaratnam AM (2008). Erythema nodosum—an association with rabies vaccination. Dermatol Online J.

[19] Corcos A (1957). Appearance of erythema nodosum at the site of a dog-bite after vaccination against rabies. [Article in French]. Bull Soc Pathol Exot Filiales.

[20] Matheis H (1971). Skin complications of smallpox vaccination. [Article in German]. Dermatologica.

[21] Thomson BJ, Nuki G (1985). Erythema nodosum following typhoid vaccination. Scott Med J.

